# Functional and prognostic implications of cardiac magnetic resonance feature tracking-derived remote myocardial strain analyses in patients following acute myocardial infarction

**DOI:** 10.1007/s00392-020-01747-1

**Published:** 2020-10-20

**Authors:** Torben Lange, Thomas Stiermaier, Sören J. Backhaus, Patricia C. Boom, Johannes T. Kowallick, Suzanne de Waha-Thiele, Joachim Lotz, Shelby Kutty, Boris Bigalke, Matthias Gutberlet, Hans-Josef Feistritzer, Steffen Desch, Gerd Hasenfuß, Holger Thiele, Ingo Eitel, Andreas Schuster

**Affiliations:** 1 Department of Cardiology and Pneumology, Göttingen Germany and German Centre for Cardiovascular Research (DZHK), Partner Site Göttingen, University Medical Center Göttingen, Georg-August University, Robert-Koch-Straße 40, Göttingen, Germany; 2 University Heart Center Lübeck, Medical Clinic II (Cardiology/Angiology/Intensive Care Medicine), University Hospital Schleswig-Holstein, Lübeck, Germany; 3 German Center for Cardiovascular Research (DZHK), Partner Site Hamburg/Kiel/Lübeck, Lübeck, Germany; 4 Institute for Diagnostic and Interventional Radiology, Göttingen Germany and German Centre for Cardiovascular Research (DZHK), Partner Site Göttingen, University Medical Center Göttingen, Georg-August University, Göttingen, Germany; 5 Helen B. Taussig Heart Center, The Johns Hopkins Hospital and School of Medicine, Baltimore, MD USA; 6 Department of Cardiology, Charité Campus Benjamin Franklin, University Medical Center Berlin, Berlin, Germany; 7 Institute of Diagnostic and Interventional Radiology, Heart Center Leipzig at University of Leipzig, Leipzig, Germany; 8 Department of Internal Medicine/Cardiology and Leipzig Heart Institute, Heart Center Leipzig at University of Leipzig, Leipzig, Germany

**Keywords:** CMR, Feature tracking, Myocardial infarction, Remote strain, Risk prediction

## Abstract

**Background:**

Cardiac magnetic resonance myocardial feature tracking (CMR-FT)-derived global strain assessments provide incremental prognostic information in patients following acute myocardial infarction (AMI). Functional analyses of the remote myocardium (RM) are scarce and whether they provide an additional prognostic value in these patients is unknown.

**Methods:**

1034 patients following acute myocardial infarction were included. CMR imaging and strain analyses as well as infarct size quantification were performed after reperfusion by primary percutaneous coronary intervention. The occurrence of major adverse cardiac events (MACE) within 12 months after the index event was defined as primary clinical endpoint.

**Results:**

Patients with MACE had significantly lower RM circumferential strain (CS) compared to those without MACE. A cutoff value for RM CS of − 25.8% best identified high-risk patients (*p* < 0.001 on log-rank testing) and impaired RM CS was a strong predictor of MACE (HR 1.05, 95% CI 1.07–1.14, *p* = 0.003). RM CS provided further risk stratification among patients considered at risk according to established CMR parameters for (1) patients with reduced left ventricular ejection fraction (LVEF) ≤ 35% (*p* = 0.038 on log-rank testing), (2) patients with reduced global circumferential strain (GCS) > −  18.3% (*p* = 0.015 on log-rank testing), and (3) patients with large microvascular obstruction ≥ 1.46% (*p* = 0.002 on log-rank testing).

**Conclusion:**

CMR-FT-derived RM CS is a useful parameter to characterize the response of the remote myocardium and allows improved stratification following AMI beyond commonly used parameters, especially of high-risk patients.

**Trial registration:**

ClinicalTrials.gov, NCT00712101 and NCT01612312

**Graphic abstract:**

Defining remote segments (R) in the presence of infarct areas (I) for the analysis of remote circumferential strain (CS). Remote CS was significantly lower in patients who suffered major adverse cardiac events (MACE) and a cutoff value for remote CS of − 25.8% best identified high-risk patients. In addition, impaired remote CS ≥ − 25.8 % (Remote −) and preserved remote CS < − 25.8 % (Remote +) enabled further risk stratification when added to established parameters like left ventricular ejection fraction (LVEF), global circumferential strain (GCS) or microvascular obstruction (MVO).
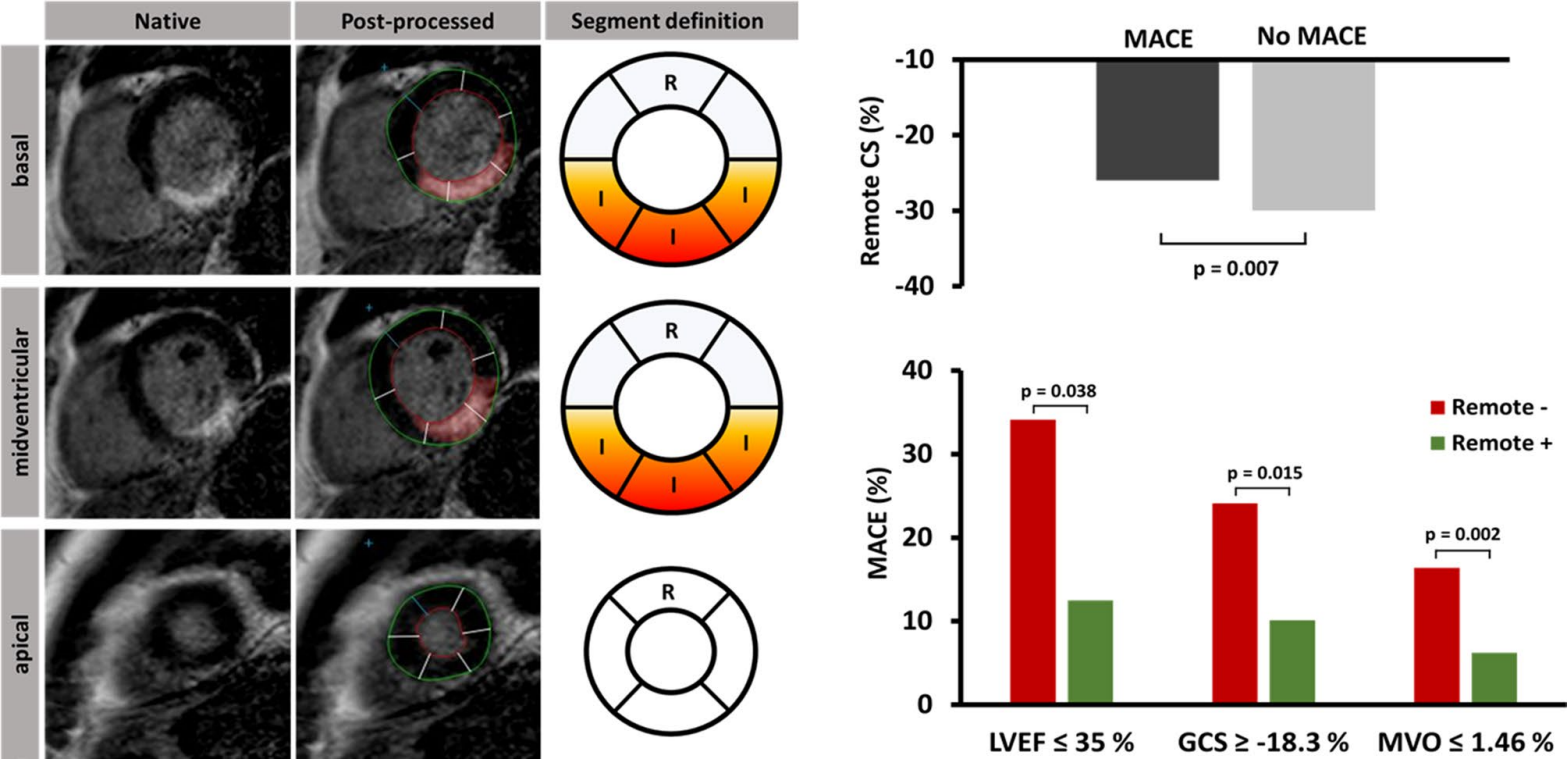

## Introduction

Cardiovascular diseases are a leading cause of premature death and require an accurate risk stratification for an optimal guideline-based treatment strategy [1]. In patients with acute myocardial infarction (AMI) various methodologies for advanced risk stratification including left ventricular ejection fraction (LVEF), global strain analyses as well as morphologic quantifications are established. Cardiac magnetic resonance (CMR) imaging has emerged as a useful and widespread modality for the assessment of both myocardial morphology and functional performance with an incremental prognostic value [2–6]. CMR feature tracking (CMR-FT)-derived strain analyses have been shown to provide useful and superior functional analyses in a wide range of cardiovascular diseases [7–10]. Tracing left-ventricular (LV) epi- and endocardial borders of the myocardium allows a comprehensive deformation assessment on a global and regional level. Additionally, CMR image analyses enable distinguishing between infarcted and remote non-infarcted myocardium (RM) based on late gadolinium enhancement (LGE) imaging [11]. While standard parameters such as LVEF and LGE-based infarct size (IS) are well established for risk stratification, it is important to remember that they measure global functional performance and extent of irreversible injury, respectively. RM function assessment on the other hand allows insights into the remaining viable myocardial tissue, which is not directly affected by a perfusion injury during AMI and may possess critical compensatory potential. Consequently, the aim of this study was to investigate RM CMR-FT-derived strain patterns and to assess their functional and prognostic implications in a large cohort of patients following AMI.

## Methods

### Study population

Data from 1034 patients, who underwent CMR imaging after undergoing primary percutaneous coronary intervention (PCI), were analyzed in this study. All patients were enrolled within the AIDA-STEMI (Abciximab Intracoronary versus intravenously Drug Application in STEMI) and TATORT-NSTEMI (Thrombus Aspiration in Thrombus Containing Culprit Lesions in NSTEMI) trials. The aims of these previous studies were on the one hand to compare the value of intravenous versus intracoronary abciximab application in STEMI patients, which did not reveal a considerable difference between both strategies (AIDA-STEMI trial), and on the other hand to examine the effect of aspiration thrombectomy versus conventional PCI, which also did not show significant differences regarding IS, MVO or clinical outcome (TATORT-NSTEMI trial). More information including detailed study protocols and results have been previously reported [12, 13]. All patients gave written informed consent before participating. Both studies were approved by all involved local ethical committees and complied with the principles of the Helsinki Declaration.

### CMR imaging protocol

All patients underwent an identical CMR protocol within 10 days after the index event. This protocol was applied on 1.5 or 3.0 T scanners at every study site and included balanced steady-state free precession sequences (SSFP) of long-axis 2- and 4-chamber views (CV) as well as short-axis (SAX) stacks. Typical SSFP sequence parameters were as follows: repetition time 3.2 ms, echo time 1.2 ms, flip angle 60°, 8 mm slice thickness in SAX. T2-weighted triple short-tau inversion recovery images (repetition time 2 RR intervals; echo time 80 ms; flip angle 90°) were generated. For the analysis of myocardial salvage, infarct size and microvascular obstruction (MVO), inversion recovery gradient echo sequences were acquired 10–20 min after a gadolinium bolus injection (0.15 mmol/kg bodyweight, repetition time 2.8 ms; echo time 1.1 ms; flip angle 15°; slice thickness 8 mm, with individually adjusted inversion times typically between 200 and 300 ms). More detailed information regarding study and scan protocols have been previously published [12, 13]. Typical contraindications to CMR applied to this study as previously detailed [13].

### CMR analysis

**Fig. 1:**
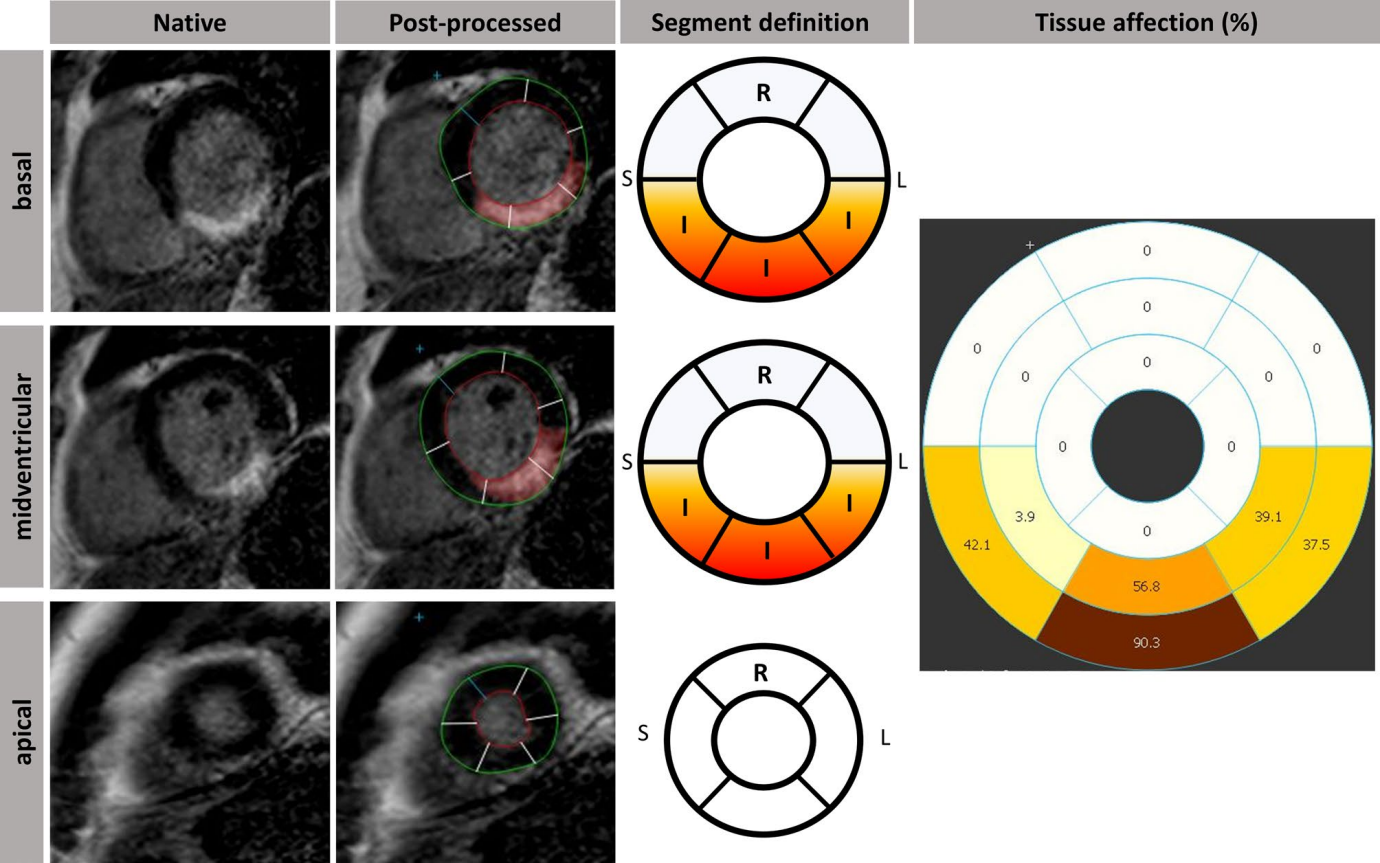
Definition of remote myocardium according to late gadolinium enhancement. Based on the AHA 16-segment model, myocardium was classified as infarcted (I) or remote (R) segments. After delineation of epi- (green) and endocardial borders (red), infarct area was plotted (red area within myocardium) and a bull’s eye displaying affected myocardial tissue (in %) was generated. The adjacent segments next to a RM segment within one layer and the adjacent segments of more basal or apical slices had to be unaffected by infarct area enhancement to define RM myocardium. *S* septal, *L* lateral

SAX segments were defined according to the American Heart Association 16-segment model [14] with segments containing LGE considered as infarct segments. RM segments were defined as unenhanced segments with one unenhanced border segment between them and infarct segments, respectively (Fig. 1). For the purposes of this paper, we defined circumferential strain (CS) in RM segments as RM CS and CS of infarct segments as infarct CS. CS of all segments is indicated as global circumferential strain (GCS). Strain analysis was performed in balanced SSFP-derived SAX stacks using dedicated evaluation software (2D CPA MR, Cardiac Performance Analysis, Version 1.1.2, TomTec Imaging Systems, Unterschleissheim, Germany). To assess SAX-derived CS, LV epi- and endocardial borders were manually tracked in basal, midventricular and apical slices to obtain global as well as regional strain values. After manual delineation of the myocardial borders at end-diastole, a semi-automated tracking algorithm was applied for tracing the contours throughout the cardiac cycle. Visual evaluations of the semi-automatically tracked contours were performed and, in case of insufficient border tracking, manual adjustments to the delineations were made with subsequent reapplication of the algorithm. All peak strain measurements are presented in percent and based on an average of three repeated and independent tracking repetitions.

For infarct area detection and quantification, dedicated post-processing software was used (QMass^®^, Version 3.1.16.0, Medis Medical Imaging Systems, Leiden, Netherlands) to manually segment LV epi- and endocardial borders in SAX orientation. LGE segments containing myocardial infarction area were defined using a full width at half maximum (FWHM) approach with excellent reproducibility [15]. The slice containing the highest signal intensity infarct area enhancement was chosen and regions of interest including infarct area and normal myocardium were marked to define thresholds for enhanced and non-enhanced myocardium. After applying semi-automated infarct area detection and performing a visual accuracy review, manual adjustments were made as required. Regions with MVO were manually included, since signal intensity is not increased in these areas.

### Clinical endpoints and outcome

The occurrence of MACE was defined as the primary clinical endpoint of this study. All-cause mortality, reinfarction or congestive heart failure associated with rehospitalization within the first year after AMI was counted as MACE. In case of multiple occurrences of MACE within one patient, a prioritization was made (death > reinfarction > congestive heart failure) with each patient only accounting for one MACE.

### Statistical analyses

Categorical parameters are presented in absolute numbers and percentages. Continuous parameters were tested for normal distribution using Shapiro–Wilk test and are reported as mean with interquartile range (IQR). For the assessment of correlations, the Spearman’s rank correlation coefficient was used. Non-parametric Mann–Whitney *U* test was used for comparisons of continuous data sets. A previously determined cutoff value for GCS (> − 18.3%) based on area under the curve analyses was used [10]. Similarly, an optimal dichotomization cutoff value for RM CS was determined by Youden’s Index. Moreover, high- and low-risk patient groups were categorized for LVEF, IS and MVO. For LVEF, subgroups with ≤ 35% and > 35% were analyzed according to established clinical practice. IS and MVO cutoff values were calculated using Youden’s Index. The Kaplan–Meier method was applied to analyze occurrence of clinical endpoints within predefined groups and log-rank testing for assessing differences between groups. Univariate hazard ratios (HRs) based on Cox proportional regression models were calculated in the context of MACE and mortality evaluation. Only variables with a *p* value < 0.05 were included in further multivariable regression calculations. Multivariable calculations comprised a stepwise approach with thresholds of 0.05 and 0.1 for *p* values to keep or remove variables, respectively. Due to significant correlations of CS values and LVEF, only one of these parameters was included in multivariable regression models in each case. All provided *p* values are two-sided with an alpha level < 0.05 considered statistically significant. IBM SPSS Statistic Software Version 24 (International Business Machines, Armonk, New York, USA) and Microsoft Excel (Microsoft, Redmond, Washington, USA) were used for all statistical calculations.

## Results

### Study population

**Fig. 2:**
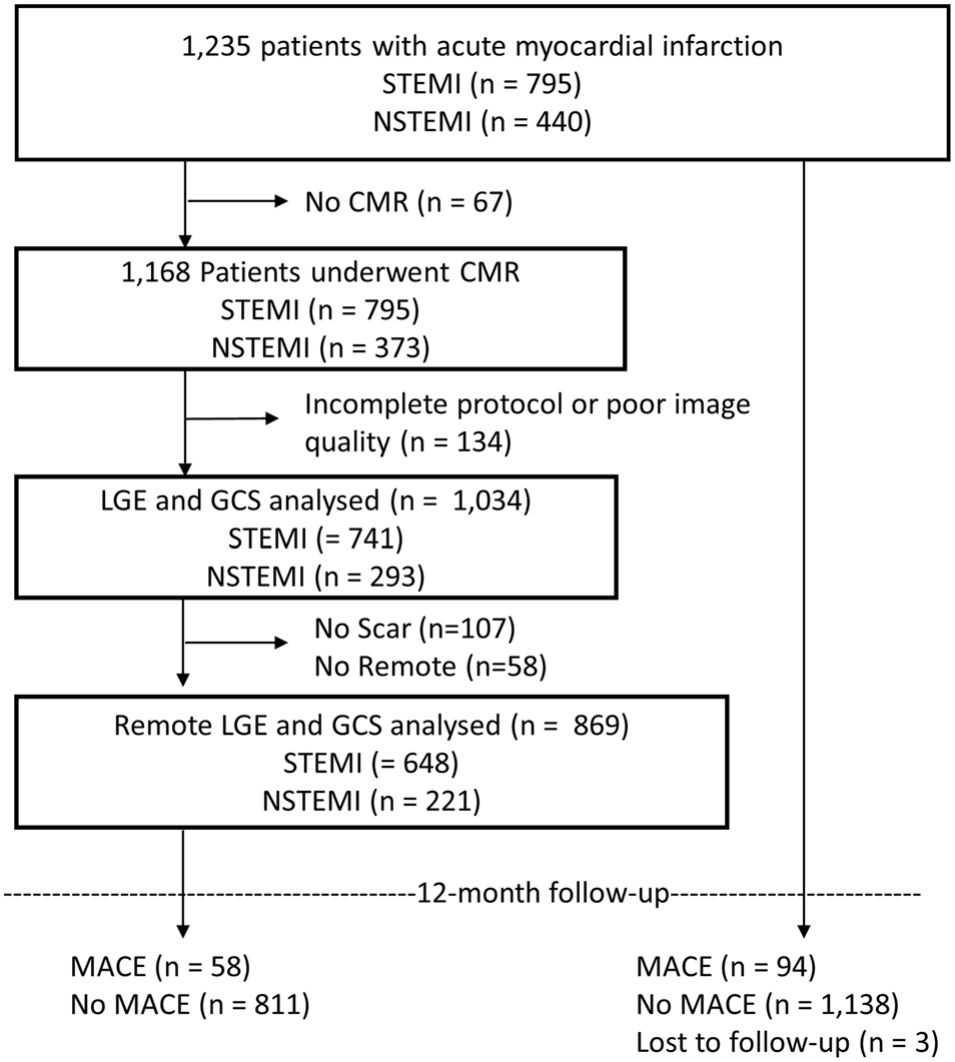
Flowchart

Among the overall cohort of 1235 patients enrolled in this CMR substudy (795 STEMI and 440 NSTEMI patients), 1034 patients with both SSFP and corresponding inversion recovery gradient SAX stacks covering base to apex were identified and included in the final analysis. 869 patients (648 STEMI and 221 NSTEMI) had evidence of infarct area on LGE imaging with a maximum extent allowing the definition of at least one RM segment (Fig. 2). Baseline characteristics of patients with RM are displayed in Table 1. The study cohort included mainly male patients (73.4%) with a median age of 64 years (IQR 52–72 years). Hypertension (*p* = 0.007) and diabetes (*p* = 0.03) were more frequent, whereas male patients (*p* = 0.008) and non-smokers (*p* = 0.013) were less frequent among patients with MACE during 1-year follow-up. Killip class on admission (*p* < 0.001) and the number of diseased vessels (*p* = 0.015) were significantly higher in patients with MACE.

**Table 1:** Baseline characteristics

Variables	All patients (*n* = 869)	MACE (*n* = 58)	No MACE (*n* = 811)	*p* value
Age	64 (52–72)	72 (61.8–77.3)	63 (52–72)	**< 0.001**
Sex (male)	638/869 (73.4)	34/58 (58.6)	604/811 (74.5)	**0.008**
Cardiovascular risk factors				
Active smoking	354/801 (44.2)	14/51 (27.5)	340/750 (45.3)	**0.013**
Hypertension	613/868 (70.6)	50/58 (86.2)	563/810 (69.5)	**0.007**
Hyperlipoproteinemia	322/864 (37.3)	19/58 (32.8)	303/806 (37.6)	0.462
Diabetes	199/868 (22.9)	20/58 (34.5)	179/810 (22.0)	**0.03**
Body mass index (kg/m^2^)	27.5 (25–30.5)	27 (25.2–31.2)	27.5 (25.0–30.5)	0.977
Previous myocardial infarction	59/869 (6.8)	5/58 (8.6)	54/811 (6.7)	0.566
Previous PCI	67/869 (7.7)	4/58 (6.9)	63/811 (7.8)	0.81
Previous CABG	14/869 (1.6)	1/58 (1.7)	13/811 (1.6)	0.944
ST-segment elevation	648/869 (74.6)	40/58 (69.0)	608/811 (75.0)	0.31
Systolic blood pressure (mmHg)	132 (117–150)	129 (104–144)	133 (118–150)	**0.024**
Diastolic blood pressure (mmHg)	80 (70–88)	75.5 (62–84)	80 (70–89)	**0.025**
Heart rate (bpm)	76 (68–86)	80 (70–96)	76 (67–86)	**0.001**
Time symptoms to balloon* (min)	180 (110–315)	191 (120–370)	180 (109–310)	0.354
Door-to-balloon time* (min)	30 (22–42)	28.5 (21.7–40)	30 (22–42)	0.609
Killip class on admission				**< 0.001**
1	772/869 (88.8)	39/58 (67.2)	733/811 (90.4)	
2	67/869 (7.7)	12/58 (20.7)	55/811 (6.8)	
3	18/869 (2.1)	3/58 (5.2)	15/811 (1.8)	
4	12/869 (1.4)	4/58 (6.9)	8/811 (1.0)	
Diseased vessels				**0.015**
1	444/869 (51.1)	23/58 (39.7)	421/811 (51.9)	
2	263/869 (30.3)	16/58 (27.6)	247/811 (30.5)	
3	162/869 (18.6)	19/58 (32.8)	143/811 (17.6)	
Affected artery				0.089
Left anterior descending	360/869 (41.4)	29/58 (50.0)	331/811 (40.8)	
Left circumflex	162/869 (18.6)	13/58 (22.4)	149/811 (18.4)	
Left main	4/869 (0.5)	0/58 (0.0)	4/811 (0.5)	
Right coronary artery	340/869 (39.1)	15/58 (25.9)	325/811 (40.1)	
Bypass graft	3/869 (0.3)	1/58 (1.7)	2/811 (0.2)	
TIMI flow grade before PCI				0.81
0	489/869 (56.3)	36/58 (62.1)	453/811 (55.9)	
1	93/869 (10.7)	5/58 (8.6)	88/811 (10.9)	
2	162/869 (18.6)	9/58 (15.5)	153/811 (18.9)	
3	125/869 (14.4)	8/58 (13.8)	117/811 (14.4)	
Stent implanted	853/869 (98.2)	57/58 (98.3)	796/811 (98.2)	0.485
TIMI flow grade after PCI				0.314
0	16/869 (1.8)	0/58 (0.0)	16/811 (2.0)	
1	19/869 (2.2)	2/58 (3.4)	17/811 (2.1)	
2	76/869 (8.7)	8/58 (13.8)	68/811 (8.4)	
3	758/869 (87.2)	48/58 (82.8)	710/811 (87.5)	
Medication				
Aspirin	869/869 (100.0)	58/58 (100.0)	811/811 (99.9)	
Clopidogrel/prasugrel/ticagrelor	869/869 (100.0)	58/58 (100.0)	811/811 (100.0)	
Beta-blocker	829/867 (95.6)	56/58 (96.6)	773/809 (95.6)	0.719
ACE-inhibitor/AT1 antagonist	813/867 (93.8)	55/58 (94.8)	758/809 (93.5)	0.73
Aldosterone antagonist	111/867 (12.8)	21/58 (36.2)	90/809 (11.1)	**< 0.001**
Statin	828/867 (95.5)	55/58 (94.8)	773/809 (95.6)	0.798
Time to MRI (days)	3 (2–4)	3 (2–4)	3 (2–4)	0.262

Data are presented as *n*/*N* (%) or median (interquartile range). For comparison of patients with MACE and no MACE, *p* values were calculated; bold numbers indicate a statistically significant difference. Mann–Whitney *U* test was used for testing continuous variables, and categorical variables were tested using Chi square test

*CABG* coronary artery bypass graft, *MACE* major adverse cardiac event, *PCI* percutaneous coronary intervention, *TIMI* thrombolysis in myocardial infarction

*Only assessed in ST-segment elevation myocardial infarction patients (*n* = 648)

### RM functional analyses

**Fig. 3:**
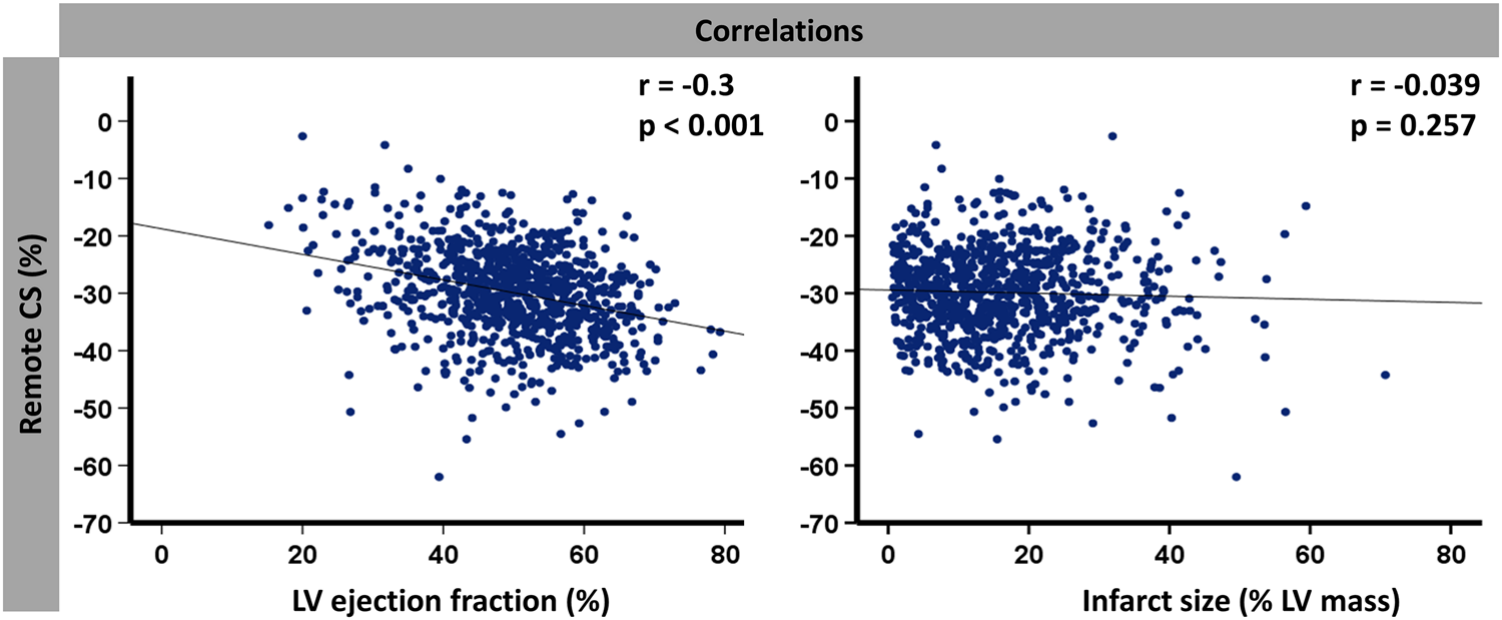
Correlation of remote CS with left ventricular (LV) ejection fraction and infarct size. Correlation of remote CS with left ventricular ejection fraction (left) and infarct size (right). *CS* global circumferential strain, *% LV* percent left ventricular mass

CS was considerably higher in RM segments compared to infarct segments and GCS (− 29.7% [− 25.0% to − 34.4%] versus − 18.7% [− 14.7% to − 23.2%] for infarct segments versus − 23.8% [− 19.4% to − 28.2%] for GCS; *p* < 0.001 for both). Similar significant differences were present between patients with and without MACE (Table 2)*.* All CS values were significantly reduced in patients with MACE during the 1-year follow-up (*p* < 0.001 for GCS and infarct CS; *p* = 0.007 for RM CS). IS and MVO were larger in patients with MACE (20.6% versus 14.2% for IS, *p* = 0.001 and 1.5% versus 0.4% for MVO, *p* = 0.01). Myocardial salvage index was higher in patients without MACE during follow-up (53.1 versus 41.8, *p* = 0.01). RM CS correlated significantly with LVEF (*p* < 0.001; *r* = − 0.3), whereas there was no significant correlation with infarct size (*p* = 0.257; *r* = − 0.039) (Fig. 3).

**Table 2:** Cardiac magnetic resonance results

	All patients	MACE	No MACE	*p* value
GCS %	− 23.8 (− 19.4 to − 28.2)	− 18.3 (− 14.4 to − 22.3)	− 24.2 (− 19.8 to − 28.6)	**< 0.001**
Remote CS %	− 29.7 (− 25.0 to − 34.4)	− 26.0 (− 20.6 to − 33.8)	− 30.0 (− 25.2 to − 34.5)	**0.007**
Infarct CS %	− 18.7 (− 14.7 to − 23.2)	− 14.5 (− 11.1 to − 19.0)	− 19.0 (− 15.1 to − 23.4)	**< 0.001**
Area at risk, % LV mass	30.4 (21.5–43.6)	33.9 (24.7–46.2)	30.2 (21.2–43.2)	0.105
Infarct size, % LV mass	14.5 (7.7–22.0)	20.6 (10.7–28.9)	14.2 (7.5–21.7)	**0.001**
Microvascular obstruction, % LV mass	0.4 (0.0–2.0)	1.5 (0.0–3.3)	0.4 (0.0–1.9)	**0.011**
Myocardial salvage, % LV mass	14.3 (8.2–23.1)	15.2 (7.8–19.7)	14.2 (8.3–23.6)	0.525
Myocardial salvage index	52.6 (33.6–68.4)	41.8 (22.8–59.7)	53.1 (35.0–68.8)	**0.012**

Values are displayed as median (interquartile range). *p* values were calculated for the comparison between patients with and without MACE using the Mann–Whitney *U* test. Numbers in bold indicate a statistical significance in difference. *CS* circumferential strain, *GCS* global circumferential strain, *MACE* major adverse cardiac events

### Prognostic implications of RM strain

**Fig. 4:**
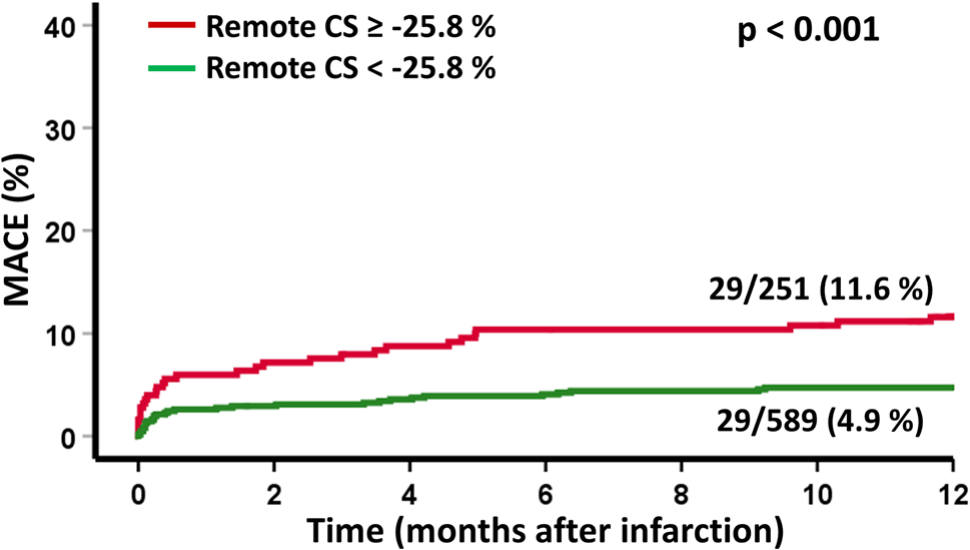
Remote CS and survival after acute myocardial infarction. Incidence of MACE (major adverse cardiac events) according to high and low remote circumferential strain (CS)

During the 1-year follow-up, 58 MACE were documented (death = 24, reinfarction = 16, congestive heart failure = 18). Using Youden’s Index, a cutoff value for RM CS of − 25.8% best classified the cohort into high- and low-risk groups according to RM function (Fig. 4). Further high- and low-risk grading according to infarct characteristics resulted in optimal cutoff values for a dichotomization with 19.2% (of LV mass) for IS and 1.46% (of LV mass) for MVO.

Univariable cox regression analyses in the overall cohort revealed an association of RM CS with MACE (HR 1.05, 95% CI 1.07–1.14, *p* = 0.003). IS (HR 1.04, 95% CI 1.02–1.06, *p* < 0.001), MVO (HR 1.1, 95% CI 1.03–1.17, *p* = 0.004) and myocardial salvage index (HR 0.99 (0.97–1.0, *p* = 0.013) were also associated with MACE (Table 3). Among patients considered at risk according to dichotomization cutoffs using established parameters, univariable regression-based hazard calculations identified RM CS as a strong predictor of MACE in patients with reduced LVEF ≤ 35% (HR 1.07 95% CI 1.02–1.13, *p* = 0.01), with reduced GCS > − 18.2% (HR 1.05 95% CI 1.02–1.09, *p* = 0.03) and with large MVO > 1.46% (HR 1.07 95% CI 1.02–1.1, *p* = 0.003). In patients with a small IS < 19.2% (HR 1.07 95% CI 1.04–1.2, *p* = 0.001), RM CS was also found to be a strong predictor of MACE. Applying multivariable regression calculations, Killip class (HR 1.49 95% CI 1.00–2.22, *p* = 0.048) and LVEF (HR 0.93, 95% CI 0.90–0.96, *p* < 0.001) remained significant after correction for all univariate significant parameters in the overall cohort (Table 3).

**Table 3:** Univariate and multivariate Cox regression analysis for prediction of MACE

Variables	Univariate hazard ratio (CI)	*p* value	Multivariate hazard ratio (CI)	*p* value
Age	1.055 (1.031–1.080)	**< 0.001**		
Sex (male)	2.0 (1.186–3.373)	**0.009**		
Smoking	0.472 (0.255–0.872)	**0.017**		
Hypertension	2.661 (1.262–5.613)	**0.01**		
Diabetes	1.804 (1.05–3.1)	**0.033**		
Systolic blood pressure (mmHg)	0.984 (0.973–0.996)	**0.008**		
Diastolic blood pressure (mmHg)	0.981 (0.962–1.0)	**0.048**		
Heart rate (bpm)	1.027 (1.013–1.041)	**< 0.001**		
Killip class on admission	2.084 (1.588–2.735)	**< 0.001**	1.49 (1.00–2.22)	**0.048**
Number of diseased vessels	1.507 (1.099–2.066)	**0.01**		
Infarct size (% LV)	1.038 (1.018–1.058)	**< 0.001**		
Microvascular obstruction (% LV)	1.096 (1.030–1.166)	**0.004**		
Myocardial salvage index	0.985 (0.974–0.997)	**0.013**		
LV ejection fraction (%)	0.933 (0.913–0.953)	**< 0.001**	0.93 (0.9–0.96)	**< 0.001**
GCS (%)	1.106 (1.07–1.143)	**< 0.001**		
Remote CS (%)	1.054 (1.068–1.173)	**0.003**		
Infarct CS (%)	1.12 (1.068–1.173)	**< 0.001**		

Bold values indicate statistical significance in difference

*CI* confidence interval, *CS* circumferential strain, *GCS* global circumferential strain, *LVEF* left ventricular ejection fraction

**Fig. 5:**
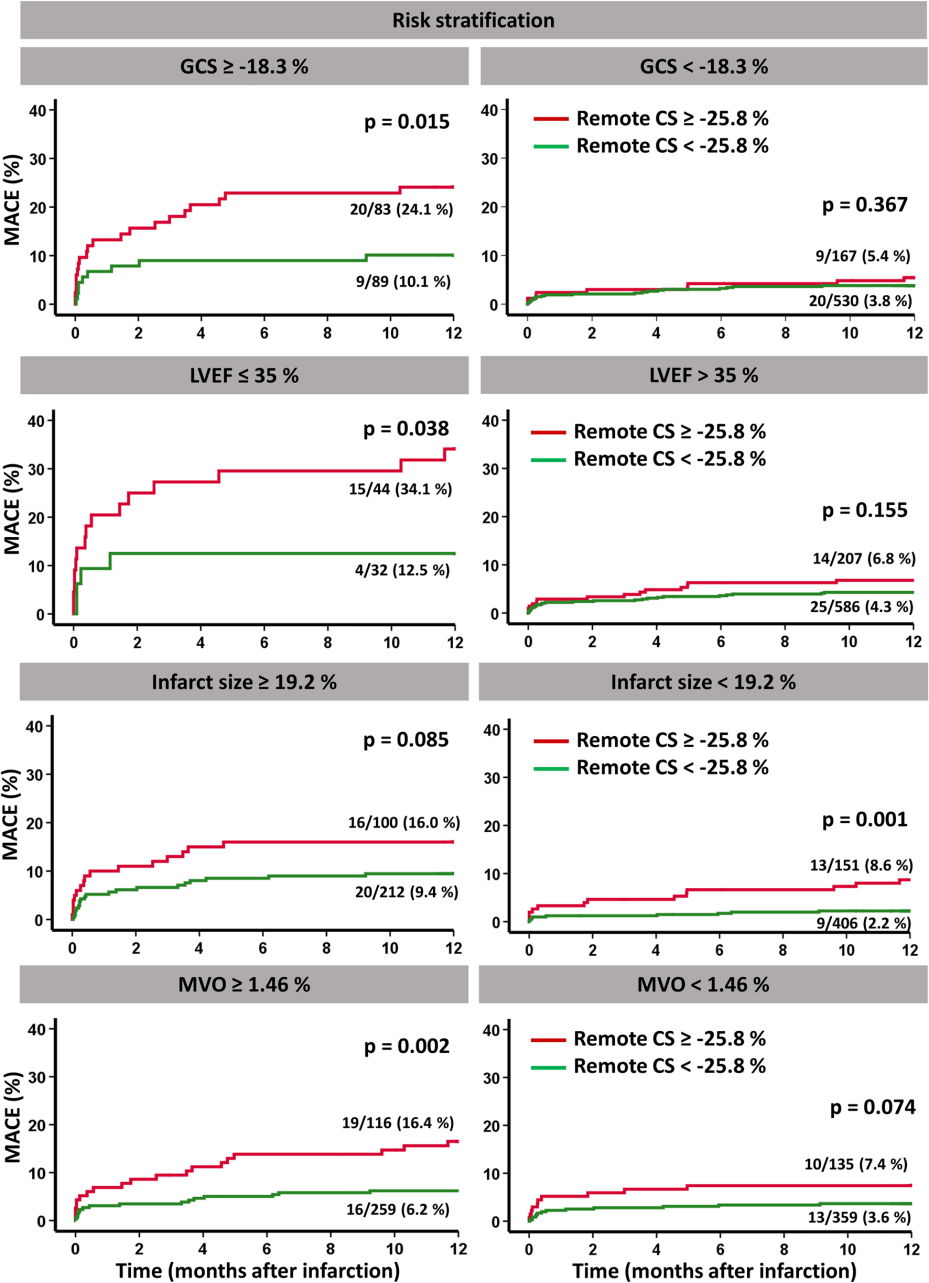
Remote circumferential strain (CS) and survival in subgroup analyses. Incidence of MACE (major adverse cardiac events) according to high and low remote circumferential strain (cutoff value − 25.8%) in subgroups of global circumferential strain (GCS), left ventricular ejection fraction (LVEF), infarct size (IS) and microvascular obstruction (MVO) dichotomized to high- and low-risk cohorts using optimal cutoff points

Furthermore, RM CS provided additional risk stratification among high-risk patients by identifying subgroups with higher jeopardy for the occurrence of MACE using Kaplan–Meier plots (log-rank test *p* = 0.015 for GCS > − 18.3%; *p* = 0.038 for LVEF ≤ 35% and *p* = 0.002 for MVO ≥ 1.46%). Regarding IS, RM CS provided additional risk stratification in small IS < 19.2% (*p* = 0.001). For both large IS and small MVO, a trend toward a statistical significance was documented (*p* = 0.085 for IS ≥ 19.2% and *p* = 0.074 for small MVO < 1.46%). Significant differences and trends are visualized by Kaplan–Meier plots in Fig. 5.

## Discussion

This study investigated CMR-based RM functional strain characteristics and their prognostic implications in a large cohort of patients following AMI.

While commonly available CMR parameters assess the extent of irreversible damage (e.g., IS or MVO) or the overall global functional performance (e.g., LVEF or global strain), we suggest focusing on the compensatory capacity of the remaining viable myocardium using remote strain CMR-FT. Indeed, RM CS strongly predicts MACE and moreover enables an extended risk stratification by better stratifying high-risk patients following AMI exceeding the information derived from irreversible damage or global performance. A failing or compensating RM may represent an important target for a more tailored therapeutic treatment using intensified heart failure medication or device therapy following AMI. Therefore, we believe that RM CS should complement the available CMR risk stratification armamentarium, allowing a more in-depth functional and prognostic characterization of the myocardium after AMI.

CMR-FT-derived strain and tissue assessments are established techniques with increasing diagnostic and prognostic implications in various cardiac diseases [16]. Considering both functional myocardial performance parameters and tissue characterization, there is a fundamental debate about which method is superior to analyze myocardial viability. Kim et al. demonstrated the prediction of improved myocardial contractility following revascularization depending on the transmurality of hyperenhanced areas on late gadolinium enhanced images [17]. On the contrary, myocardial functional performance, especially of the remaining viable tissue, was found to be superior to IS quantification by Wellnhofer et al. [18]. These landmark studies have implications beyond the diagnosis of viable myocardium and prediction of functional recovery in chronic coronary artery disease [19] and it is interesting to interpret their findings in the context of AMI. A combination of regional functional and morphologic CMR parameters provides a more in-depth characterization of AMI-related injury which cannot be entirely captured with just injury (e.g., IS, MVO or myocardial salvage) or function (e.g., LVEF or GCS) quantification.

While global strain values or IS only indirectly represents the compensatory capacity of the post-infarct heart, functional remote area analyses may provide the tools to identify patients at high risk and patients that are likely to overcome the acute event [20]. Based on our data showing distinct differences between RM, infarct and global strain as well as the association of high remote strain with favorable outcome supports the assumption of a compensatory role of RM strain following AMI.

There is a clear need to improve the identification of patients at risk and recent evidence suggests that current approaches such as an LVEF cutoff of 35% for ICD device therapy may not be sufficient for this purpose [21]. RM CS, especially, allowed further risk stratification in patients that are already considered high-risk based on additional conventional CMR parameters including reduced LVEF, GCS and MVO. However, RM CS did not provide significantly better discrimination of patients with large IS. This may be explained by the definition of RM CS requiring non-infarcted segments between infarcted and remote segments which limits the number of remote segments particularly in patients having large IS. Notwithstanding it is important to note that there was no association of RM CS and IS, suggesting that the compensation of AMI by remote function is a distinct pathophysiological feature, which is irrespective of the size of myocardial injury.

Considering RM as a potential target for treatment strategies, several studies have demonstrated the beneficiary effects of therapeutic approaches, for example inducing a reduced heart rate, [22], anti-apoptotic effects in RM [23] or remodeling prevention via an angiotensin–AT1-receptor-dependent mechanism [24]. Further explorations of RM strain characteristics and their features as targets of pharmacological treatment are necessary in future studies.

On a technical level, RM CS analysis can be easily performed within routinely acquired standard SSFP images using routine post-processing. Hence, no extra CMR sequences are required and data analyses can be easily conducted with clinically approved software solutions. Especially patients having reduced cardiac performance and being classified to high-risk groups according to commonly used parameters can be assessed precisely and efficiently. Furthermore, since fully automatic strain and function quantification with dedicated post-processing software is feasible, these time-saving and highly reproducible methods might additionally facilitate the implementation of RM strain assessment in clinical routine [25–28].

### Limitations

Several study sites performed CMR imaging using different vendors. Nevertheless, all centers followed the same study protocol and image post-processing was performed centrally in an experienced core laboratory. Regional strain reproducibility is generally lower compared to global values [29]. Nevertheless, CS has the highest reproducibility among myocardial deformation indices on a regional level [30] and was found to correlate best with LGE [26] and therefore might represent the most valid strain parameter for RM assessment. When defining RM segments, the percentage of infarct area in infarcted segments is not considered. Effects of a minimally affected infarct segment might be different compared to a completely infarcted segment. However, defining RM in accordance with the AHA 16-segment model allows an objective and standardized segment classification.

## Conclusion

CMR-FT-derived RM CS represents a useful parameter for myocardial functional performance analysis and risk assessment in a large cohort of patients following AMI. RM CS analysis allows an extended risk stratification by identifying additional high-risk groups beyond commonly used parameters and therefore might allow better patient selection for optimized treatment strategies with subsequently improved outcome following AMI in the future.

## Data Availability

The data that support the findings of this study are available from the corresponding author on reasonable request.

## Code availability

2D CPA MR, Cardiac Performance Analysis, Version 1.1.2, TomTec Imaging Systems, Unterschleissheim, Germany.
